# Readmission rates of South Korean psychiatric inpatients by inpatient volumes per psychiatrist

**DOI:** 10.1186/s12888-016-0804-y

**Published:** 2016-04-08

**Authors:** Kyu-Tae Han, Seo Yoon Lee, Sun Jung Kim, Myung-Il Hahm, Sung-In Jang, Seung Ju Kim, Woorim Kim, Eun-Cheol Park

**Affiliations:** Department of Public Health, Graduate School, Yonsei University, Seoul, Republic of Korea; Institute of Health Services Research, Yonsei University College of Medicine, Seoul, Republic of Korea; Department of Health Policy and Management, Graduate School of Public Health, Yonsei University, Seoul, Republic of Korea; Department of Health Administration and Management, Soonchunhyang University, Asan, Republic of Korea; Department of Preventive Medicine, Yonsei University College of Medicine, 50 Yonsei-ro, Seodaemun-gu, Seoul 120-752 Republic of Korea

**Keywords:** Patient volume, Quality of care, Readmission, Psychiatric care

## Abstract

**Background:**

Readmission rates of psychiatric inpatients are higher in South Korea than other Organization for Economic Co-operation and Development (OECD) countries. In addition, the solution for readmission control is deficient based on the characteristics of the South Korean National Health Insurance (NHI) system. Therefore, it is necessary to identify ways to reduce psychiatric inpatient readmissions. This study investigated the relationship between inpatient volume per psychiatrist and the readmission rate of psychiatric inpatients in South Korea.

**Method:**

We used NHI claim data (*N* = 37,796) from 53 hospitals to analyze readmission within 30 days for five diagnosis (organic mental disorders, mental and behavioral disorders due to psychoactive substance use, schizophrenia, mood disorders, neurotic disorders, and stress-related and somatoform disorders) between 2010 and 2013. We performed *χ*2 and analysis of variance tests to investigate associations between patient and hospital-level variables and readmission within 30 days. Finally, generalized estimating equation (GEE) models were analyzed to examine possible associations with readmission.

**Results:**

Readmissions within 30 days accounted for 1,598 (4.5 %) claims. Multilevel analysis demonstrated that inpatient volume per psychiatrist were inversely related with readmission within 30 days (low odds ratio [OR]: 0.38, 95 % confidence interval [CI]: 0.28–0.51; mid-low OR: 0.48, 95 % CI: 0.36–0.63; mid-high OR: 0.55, 95 % CI: 0.44–0.69; Q4 = ref). The subgroup analysis by diagnosis revealed that both “schizophrenia, schizotypal, and delusional disorders” and “mood disorders” had inverse relationships with readmission risk for all volume groups.

**Conclusions:**

We observed an inverse association between inpatient volume per psychiatrist and the 30-day readmission rate of psychiatric inpatients, suggesting that it could be a useful quality indicator in mental health care.

## Background

In South Korea, readmission is a key issue for controlling health care costs from increasing. However, readmission rates have gradually increased, especially for mental health care. It adversely affects patients and increases the cost burden on National Health Insurance (NHI) systems. Thus, readmission is considered a quality indicator for inpatient services [1]. Based on the Organization for Economic Co-operation and Development (OECD) Health at a Glance 2013 data, the readmission rate of psychiatric inpatients in South Korea was greater than those in other OECD countries (readmissions for schizophrenia in South Korea, 2011: 19.4 %; OECD average: 12.3 %) [2].

Many health care professionals and researchers have investigated ways to reduce inpatient readmission. These studies reported that risk of readmission in this patient group is positively associated with a history of previous readmission, length of stay (LOS), and comorbidity, but were inversely associated with substance abuse, and hospital volume [3–9]. Among those, hospital volume was generally considered to be associated with better health care quality. Regarding the volume-outcome relationship, higher volume could be helpful in the improvement of skills and the outcomes were improved [10, 11]. However, excessive volume may be a cause of overload in hospital staffing, and most publications that describe a volume-outcome relationship only analyzed health care requiring surgical procedures; they did not assess psychiatric health care. The characteristics of medical treatment in mental health services are different from other specialties. Given that psychiatrist treatment largely involves patient interview sessions rather than surgical procedures, such treatments would differently affect psychiatrist`s workload compared to other specialty areas. Consequently, the reduction in the quality of care for psychiatric disorder can be caused by such association. Hence readmission, a healthcare quality indicator, can increase because patients are unable to receive appropriate treatment due to a reduction in the quality of care [12–15]. This suggests that the volume-outcome relationship is not applicable to all areas of medical treatment.

Although the problem of psychiatric readmission rates in South Korea was worse than other countries, since South Korea had FFS (fee for service) payment system for psychiatric disorder, the solution for control of the readmission of psychiatric inpatients were deficient by the characteristics of NHI system in South Korea [16, 17]. To the best of our knowledge, no studies about patient volume per psychiatrist have been conducted in South Korea. Therefore, it is necessary to investigate how patient volumes per psychiatrist affect psychiatric inpatient admissions in order to improve outcomes and reduce medical costs.

## Methods

### Study population

**Fig. 1:**
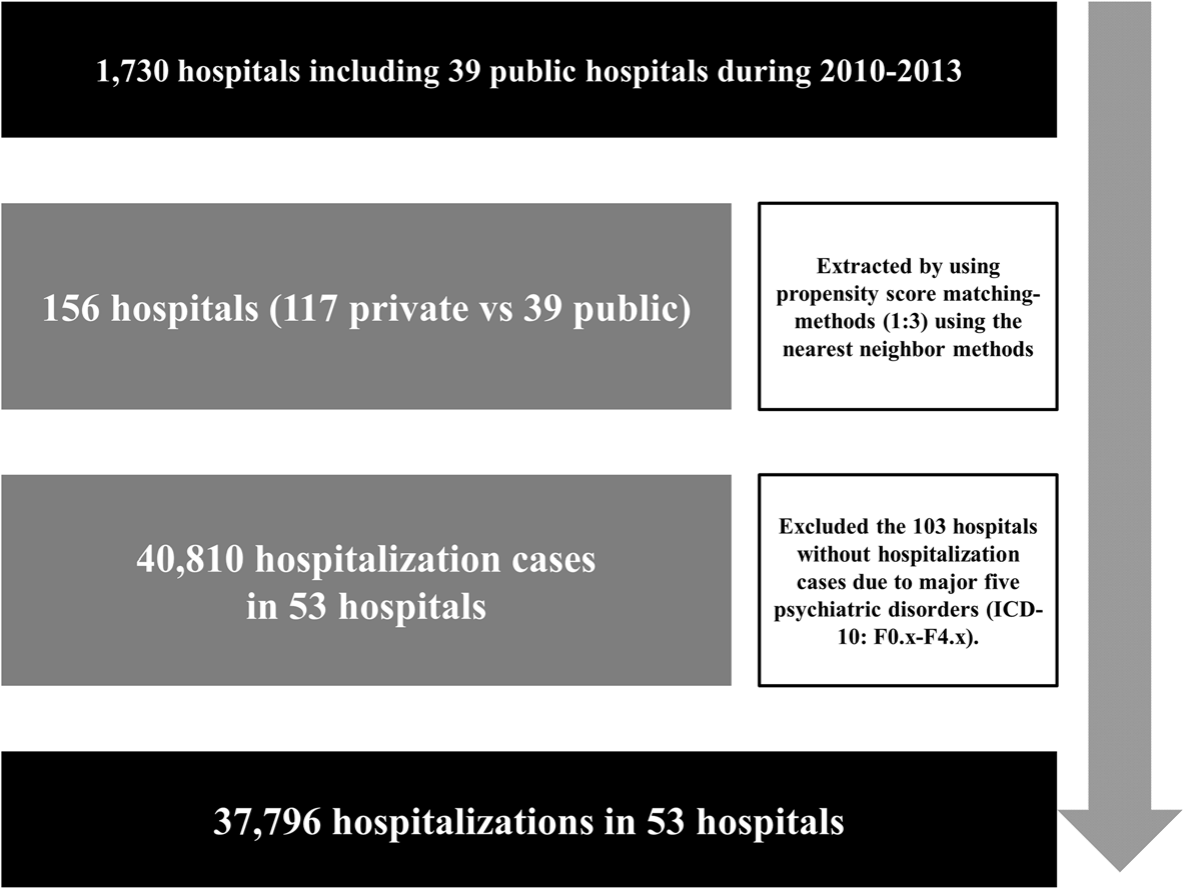
Selection of study population

There were about 1,730 hospitals including 39 public hospitals during 2010–2013 in South Korea. We projected that this might cause baseline imbalances due to differences in hospital characteristics because the number of public hospitals was only about 2.3 % among the total number of hospitals in South Korea. To reduce bias caused by the observed covariates and to deal with the usual baseline imbalances across hospitals, the data used in this study only included 156 hospitals (117 private vs 39 public) that were extracted using the propensity score matching-methods (1:3), conducted based on the nearest neighbor methods while adjusting for hospital characteristics including hospital location, nursing staffing level, number of total beds, number of intensive care unit beds, number of emergency room beds, and number of doctors [18]. Among 156 hospitals, we only analyzed hospitalization cases of the five most frequent diagnostic categories among overall psychiatric disorder that classified diagnoses according to International Classification of Diseases groupings (ICD-10: F0.x-F4.x) to reflect specific clinical mechanisms as follows: “organic, including symptomatic, mental disorders (F0.x)”, “mental and behavioral disorders due to psychoactive substance use (F1.x)”, “schizophrenia, schizotypal, and delusional disorders (F2.x)”, “mood disorders (F3.x)”, and “neurotic, stress-related and somatoform disorders (F4.x)”. Therefore, we excluded the 103 hospitals without hospitalization cases for the major five psychiatric disorders. Then, we excluded 3,014 cases with missing values for the variables of interest. Finally, we used NHI claim data from 53 hospitals to analyze readmission rates of psychiatric inpatients within 30 days of discharge. These data were collected between 2010 and 2013 and included 37,796 hospitalizations in 53 hospitals. The unit of analysis was hospitalization rather than patient (Fig. 1).

### Ethics statement

Out study utilized secondary data reported on the aggregate level, and each case in our dataset was converted to random number for preventing identification. Therefore, it was not necessary to obtain research ethics approval.

### Variables

The outcome variable in this study was readmission within 30 days due to same diagnosis after discharge for psychiatric disorders, were included in this study (F0.x-F4.x). Each patient’s first discharge in a given calendar date was considered the index discharge, and readmissions due to same diagnosis within 30 calendar days from the index discharge were defined as readmission.

The primary variable of interest in relation to readmission within 30 days of discharge was inpatient volume per psychiatrist, which was defined as the sum of LOS for all mental disorders per hospital/number of psychiatrists per hospital [19]. We assumed that there were substantial differences in risk of readmission, the outcome variable, by inpatient volume per psychiatrist in a U-shape curve. This is because higher volume in hospital could have a positive role on patient`s outcomes by increasing experiences and developing medical skills in doctors based on a volume-outcome relationship, but excessive patient volume also could negatively affect patient outcomes [12, 20, 21]. Thus, inpatient volume per psychiatrist was categorized into quartiles for analysis to reflect differences by variation of inpatient volume per psychiatrist (Q1 = low inpatient volume per psychiatrist; < 1282.0 days, Q2 = mid-low inpatient volume per psychiatrist; 1282.0–1927.2 days, Q3 = mid-high inpatient volume per psychiatrist; 1927.3–3494.9 days, Q4 = high inpatient volume per psychiatrist; more than 3495.0 days per 1 psychiatrist for 1 year) [15, 22].

We adjusted for both patient and hospital-level variables when analyzing the relationship between inpatient volume per psychiatrist and psychiatric inpatient readmission. To adjust patient demographic and clinical characteristics, patient-level variables included in the analysis were: diagnosis, LOS, sex, age, and year. Age was classified into 10-years intervals. Diagnoses were classified using ICD-10 codes F0.x-F4.x to reflect specific clinical mechanisms. LOS was included to reflect severity of each patient. We categorized it based on its median value in our study (median: 14 days, interquartile range [IQR]: 25 days) [23].

Hospital-level variables encompassed both human resource variables and structural characteristics: numbers of psychiatrists, pharmacists, nurses, and beds; proportion and occupancy rate of psychiatric beds; teaching and ownership status; and type of medical institution. The proportion of psychiatric beds was defined as a percentage of the number of psychiatric beds among the number of total beds.

The occupancy rate of psychiatric beds was calculated as the total number of inpatients days due to psychiatric disorder (ICD-10: Fx.x) for each year divided by the number of available psychiatric beds for each year [24]. This indicator was used to consider situations related to economic issues which could affect to readmission for profit in each hospital, and was calculated as shown in the following equations:

$$ \begin{array}{l} Occupancy\  rate\  of\  psychiatric\  beds = \Big( Sum\  of\  inpatient\  days\  of\  care\ for\ \\ {} psychiatric\  disorders\ for\ 1\  year/ total\  psychiatric\  beds \times 365\Big) \times 100\end{array} $$

In addition, teaching status, ownership status, and type of medical institution were included to reflect the differences by hospital structural characteristics [25, 26].

### Statistical analysis

We first examined the distribution of each categorical variable by examining frequencies and percentages and performing *χ*^2^ tests to investigate associations with psychiatric inpatient readmission within 30 days. These analyses were performed for both patient- and hospital-level variables. Next, analyses of variances (ANOVAs) were carried out to compare the average values and standard deviations for continuous hospital-level variables. Third, to examine associations with readmission within 30 days after discharge for 5 types of psychiatric disorder, we performed logistic regression analysis using generalized estimating equation (GEE) model with link logit including both inpatient- and hospital-level variables, because the data used in this study was hierarchically structured and had binary outcome variables. This GEE model assumed that with proper distributions for each hospitalization case while taking into account the correlation among hospitalization cases within the hospitals. In this study, the correlation structure was modeled as exchangeable correlation structure [27, 28]. Finally, subgroup analyses for associations with readmission within 30 days were also performed according to diagnosis. All statistical analyses were performed using SAS statistical software version 9.2 (SAS Institute Inc., Cary, NC). All P-values were two-sided and considered significant at P < 0.05.

## Results

The data used in this analysis included 35,884 hospitalizations. Among them, 1,598 (4.5 %) were psychiatric inpatient readmissions within 30 days. Table 1 shows univariate associations between various patient-level variables and readmission within 30 days. In terms of diagnoses, “schizophrenia, schizotypal, and delusional disorders” were the most frequent causes of readmission within 30 days (10.5 %) compared to other mental disorders. Patients hospitalized for more than 14 days had higher readmission rates than those with shorter hospitalizations (<14 days: 1.8 %, ≥14 days: 6.6 %). The readmission rate for males was higher than that for females (males: 6.5 %, females: 2.8 %). Finally, readmission rates gradually increased over time. The high inpatient volume group’s psychiatric inpatients had higher readmission rates than the other inpatient volume groups (low: 1.7 %, mid-low: 1.8 %, mid-high: 2.6 %, 10 high: 11.5 %).

**Table 1:** Univariate associations between readmission within 30 days of discharge and various patient-level variables

Variables	Total (*N* = 35,884)					Low inpatient volumes per psychiatrist (*n* = 8,969)					Mid-low inpatient volumes per psychiatrist (*n* = 8,909)					Mid-high inpatient volumes per psychiatrist (*n* = 9,186)					High inpatient volumes per psychiatrist (*n* = 9,042)				
	Readmission		No readmission		*P*-value	Readmission		No readmission		*P*-value	Readmission		No readmission		*P*-value	Readmission		No readmission		*P*-value	Readmission		No readmission		*P*-value
	N	%	N	%		N	%	N	%		N	%	N	%		N	%	N	%		N	%	N	%	
Diagnosis																									
Organic, including symptomatic, mental disorders	121	3.2	3,690	96.8	<.0001	6	0.5	1,173	99.5	<.0001	11	1.5	738	98.5	0.1191	25	3.0	815	97.0	<.0001	79	7.6	964	92.4	<.0001
Mental and behavioral disorders due to psychoactive substance use	288	4.7	5,875	95.3		30	1.7	1,713	98.3		40	2.5	1,542	97.5		38	3.2	1,143	96.8		180	10.9	1,477	89.1	
Schizophrenia, schizotypal, and delusional disorders	797	10.5	6,802	89.5		37	3.1	1,148	96.9		35	2.0	1,759	98.0		94	4.7	1,918	95.3		631	24.2	1,977	75.8	
Mood disorders	274	2.1	12,603	97.9		50	1.6	3,067	98.4		50	1.5	3,329	98.5		63	1.8	3,514	98.2		111	4.0	2,693	96.0	
Neurotic, stress-related, and somatoform disorders	118	2.2	5,316	97.8		30	1.7	1,715	98.3		26	1.9	1,379	98.1		23	1.7	1,331	98.3		39	4.2	891	95.8	
Length of stay (days)																									
< 14	292	1.8	15,895	98.2	<.0001	74	1.4	5,106	98.6	0.0177	74	1.8	3,927	98.2	0.8425	64	1.8	3,452	98.2	<.0001	80	2.3	3,410	97.7	<.0001
≥ 14	1,306	6.6	18,391	93.4		79	2.1	3,710	97.9		88	1.8	4,820	98.2		179	3.3	5,269	96.7		960	17.3	4,592	82.7	
Sex																									
Male	1,029	6.5	14,807	93.5	<.0001	70	1.9	3,610	98.1	0.2311	74	2.0	3,660	98.0	0.3268	157	4.3	3,518	95.7	0.3268	728	15.3	4,019	84.7	<.0001
Female	569	2.8	19,479	97.2		83	1.6	5,206	98.4		88	1.7	5,087	98.3		86	1.6	5,203	98.4		312	7.3	3,983	92.7	
Age (years)																									
≤ 29	124	2.0	6,150	98.0	<.0001	28	1.7	1,593	98.3	0.0156	27	1.5	1,828	98.5	0.1145	31	1.9	1,582	98.1	<.0001	38	3.2	1,147	96.8	<.0001
30–39	228	4.4	4,919	95.6		31	2.7	1,120	97.3		32	2.3	1,375	97.7		51	4.0	1,216	96.0		114	8.6	1,208	91.4	
40–49	369	5.8	6,029	94.2		29	2.0	1,444	98.0		34	2.1	1,564	97.9		35	2.3	1,461	97.7		271	14.8	1,560	85.2	
50–59	345	4.9	6,701	95.1		25	1.4	1,736	98.6		36	2.2	1,635	97.8		52	2.9	1,745	97.1		232	12.8	1,585	87.2	
60–69	345	6.5	4,946	93.5		25	1.8	1,333	98.2		20	1.7	1,136	98.3		52	3.8	1,317	96.2		248	17.6	1,160	82.4	
70–79	187	3.3	5,541	96.7		15	0.9	1,590	99.1		13	1.1	1,209	98.9		22	1.5	1,400	98.5		137	9.3	1,342	90.7	
Year																									
2010	120	2.3	5,124	97.7	<.0001	17	0.7	2,396	99.3	<.0001	27	1.4	1,837	98.6	<.0001	8	1.4	561	98.6	<.0001	68	17.1	330	82.9	<.0001
2011	363	3.1	11,385	96.9		16	1.0	1,618	99.0		31	1.1	2,762	98.9		46	1.1	4,224	98.9		270	7.3	3,408	92.7	
2012	702	5.4	12,242	94.6		15	1.0	1,442	99.0		43	1.7	2,494	98.3		66	1.8	3,597	98.2		578	12.4	4,082	87.6	
2013	413	6.9	5,535	93.1		105	3.0	3,360	97.0		61	3.6	1,654	96.4		123	18.0	561	82.0		124	40.5	182	59.5	
Total	1,598	4.5	34,286	95.5		153	1.7	8,816	98.3		162	1.8	8,747	98.2		243	2.6	8,943	97.4		1,040	11.5	8,002	88.5	

Table 2 shows the distribution of hospital-level variables. The average number of psychiatrists, nurses, and pharmacists were 2.9 (SD: 1.6), 8.6 (SD: 9.1), and 224.4 (SD: 165.7), respectively. The mean number of total beds was 461.5 (SD: 208.8), and the average occupancy rate of psychiatric beds was 20.1 % (SD: 47.2). There were fewer nonteaching hospitals in our sample (*n* = 17) than teaching hospitals (*n* = 36), and there were more private hospitals (*n* = 39) than public hospitals (*n* = 14). Finally, there were more general hospitals (*n* = 48) than hospitals (*n* = 5).

**Table 2:** Hospital-level characteristics

	n/Mean	%/SD
Number of psychiatrists	2.9	±1.6
Number of pharmacists	8.6	±9.1
Number of nurses	222.4	±165.7
Number of beds	461.5	±208.8
Proportion of psychiatric beds (%)	8.3	±14.2
Psychiatric bed occupancy rate (%)	20.1	±47.2
Teaching status		
Nonteaching hospital	17	32.1 %
Teaching hospital	36	67.9 %
Ownership		
Public	14	26.4 %
Private	39	73.6 %
Type of medical institution		
Hospital	5	9.4 %
General hospital	48	90.6 %
Total	53	100.0

A logistic regression analysis using GEE model considering both patient- and hospital-level variables revealed that patients with diagnoses of “schizophrenia, schizotypal, and delusional disorders (ICD-10: F2.x)” had a higher risk of readmission within 30 days compared to other mental disorders (odds ratio [OR]: 3.86, 95 % confidence interval [CI]: 2.78–5.36). Patients hospitalized for more than 14 days had a lower risk of readmission within 30 days than those hospitalized for shorter periods (OR: 0.40, 95 % CI: 0.33–0.49). Females had a lower risk of readmission than males (OR: 0.63, 95 % CI: 0.54–0.74). Over the 4 years of data included in the study, the risk for readmission gradually increased. In terms of hospital-level variables, inpatients at hospitals with low inpatient volume per psychiatrist had low risk of readmission than those with high inpatient volume (low OR: 0.38, 95 % CI: 0.28–0.51; mid-low OR: 0.48, 95 % CI: 0.36–0.63; mid-high OR: 0.55, 95 % CI: 0.44–0.69; Q4 = ref). In addition, risk of readmission had a positive trend with inpatient volume (P for trend < .001). Patients treated at hospitals with more psychiatrists or more pharmacists had a lower risk of readmission within 30 days (number of psychiatrists OR: 0.87, 95 % CI: 0.80–0.95 per psychiatrist; number of pharmacists OR: 0.94, 95 % CI: 0.92–0.96 per pharmacist). Similarly, the number of nurses was associated with a lower risk of 30-day readmission (number of nurses OR: 0.98, 95 % CI: 0.96–0.99 per 10 nurses). A higher number of total beds and greater proportion of psychiatric beds were both associated with a higher risk of readmission within 30 days (number of beds OR: 1.48, 95 % CI: 1.35–1.63 per 100 increase in the number of beds; proportion of psychiatric beds OR: 1.08, 95 % CI: 1.02–1.15 per 10 % increase in the proportion of psychiatric beds). With regard to structural characteristics, patients at private hospitals had a higher readmission risk than those at public hospitals (OR: 3.00, 95 % CI: 2.41–3.74). In contrast, patients treated at general hospitals had a lower risk of readmission within 30 days than patients at hospitals among types of medical institution (OR: 0.35, 95 % CI: 0.27–0.47) (Table 3).

**Table 3:** Factors associated with readmission within 30 days of discharge for 5 types of psychiatric disorder, derived from a GEE model

	OR	95 % CI	
Patient-level			
Diagnosis			
Organic, including symptomatic, mental disorders	1.00	–	–
Mental and behavioral disorders due to psychoactive substance use	1.56	1.10	2.22
Schizophrenia, schizotypal, and delusional disorders	3.86	2.78	5.36
Mood disorders	1.10	0.79	1.54
Neurotic, stress-related, and somatoform disorders	1.35	0.92	2.00
Length of stay (days)			
< 14	1.00	–	–
≥ 14	0.40	0.33	0.49
Sex			
Male	1.00	–	–
Female	0.63	0.54	0.74
Age (years)			
≤ 29	1.00	–	–
30–39	1.37	1.00	1.86
40–49	1.56	1.17	2.07
50–59	1.82	1.36	2.43
60–69	2.27	1.69	3.05
70–79	1.28	0.91	1.81
Year			
2010	1.00	–	–
2011	1.26	0.92	1.74
2012	1.97	1.45	2.68
2013	4.61	3.35	6.35
Hospital-level			
Inpatient volumes per psychiatrist			
Low	0.38	0.28	0.51
Mid-low	0.48	0.36	0.63
Mid-high	0.55	0.44	0.69
High	1.00	–	–
Number of psychiatrists	0.87	0.80	0.95
Number of pharmacists	0.94	0.92	0.96
Number of nurses (per 10 nurse increase)	0.98	0.96	0.99
Number of beds (per 100 bed increase)	1.48	1.35	1.63
Proportion of psychiatric beds (per 10 % increase)	1.08	1.02	1.15
Psychiatric bed occupancy rate (per 10 % increase)	0.99	0.97	1.00
Teaching status			
Nonteaching hospital	1.39	1.00	1.91
Teaching hospital	1.00	–	–
Ownership			
Public	1.00	–	–
Private	3.00	2.41	3.74
Type of medical institution			
Hospital	1.00	–	–
General hospital	0.35	0.27	0.47

*Abbreviations*: *CI* confidence interval, *OR* odds ratio

**Fig. 2:**
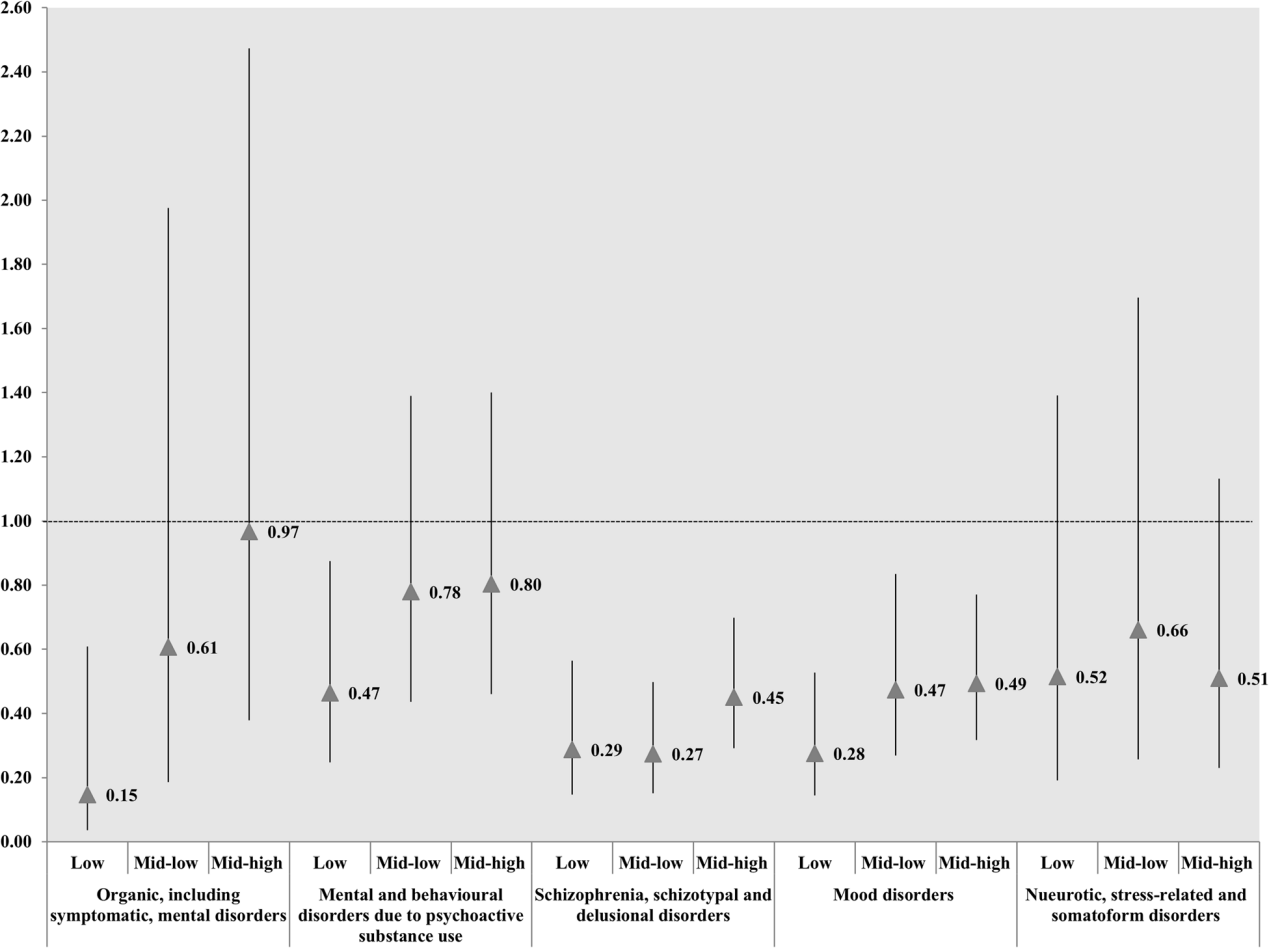
Odds ratios for inpatient volumes per psychiatrist associated with readmission within 30 days of discharge, stratified by diagnosis * High inpatient volumes per psychiatrist was reference group. † the OR is marked as triangle point; and results were statistically significant if each bar as marked to SD is not reached the cutoff line in 1.00

We performed an additional analysis investigating the association between inpatient volume per psychiatrist and patient readmission within 30 days after stratifying by each diagnosis. We observed a general positive association between inpatient volume per psychiatrist and readmission risk in all diagnosis groups. In particular, higher inpatient volume per psychiatrist group had higher risk for readmission due to “schizophrenia, schizotypal, and delusional disorders” and “mood disorders” diagnostic groups (“schizophrenia, schizotypal, and delusional disorders”: low OR: 0.29, 95 % CI: 0.15–0.56; mid-low OR: 0.27, 95 % CI: 0.15–0.50; mid-high OR: 0.45, 95 % CI: 0.29–0.70, Q4 = ref) (“mood disorders”: low OR: 0.28, 95 % CI: 0.14–0.53; mid-low OR: 0.47, 95 % CI: 0.27–0.84; mid-high OR: 0.49, 95 % CI: 0.32–0.77, Q4 = ref) (Fig. 2).

## Discussion

Readmission is considered a quality indicator for treatment, and high levels of readmission increase cost. Many studies have been conducted to assess the reasons for readmission, which is a major contributor to health care cost. In particular, the readmission of psychiatric inpatients in South Korea was higher than that of other OECD countries [2]. In the present study, we analyzed the relationship between readmission within 30 days of discharge for psychiatric disorders and inpatient volume per psychiatrist, hospital characteristics, and human resource variables. We found evidence that inpatient volume per psychiatrist was positively associated with psychiatric inpatient readmission within 30 days (Q1 OR: 0.38, 95 % CI: 0.29–0.50; Q2 OR: 0.44, 95 % CI: 0.34–0.59; Q3 OR: 0.57, 95 % CI: 0.46–0.71; Q4 = ref), not volume-outcome relationship in any level. Generally, psychiatrists with low inpatient volume (less than 1282.0 days for 1 year) had better quality in psychiatric inpatient care except patient with “neurotic, stress-related, and somatoform disorders”. In particular, those relationships were statistically significant in “schizophrenia, schizotypal, and delusional disorders” and “mood disorders”. However, in other diagnostic groups, there were just positive trends rather than statistically significant associations. It may be caused by a lack of statistical power.

Many previous studies that examined volume-outcome relationships found that better health care outcomes were associated with many patient volumes in hospitals [29, 30]. However, such studies were performed by subjecting surgical patients in surgical field. In those health care fields including surgery, physicians with high inpatient volume were found to have higher skill levels. On the other hand, psychiatrist treatment largely involves patient interview session rather than surgical procedure. Therefore, a high patient volume could cause mental fatigue for psychiatrists [31–34]. Higher level of psychiatrist burnout could contribute to poorer mental health care outcome. Although previous study in nonsurgical field demonstrated that high volume was positively associated with the risk in adverse events as readmission, our findings focused to psychiatric inpatient care in contrast with those of previous studies [35].

A few studies outside of South Korea have assessed the relationship between inpatient volume per psychiatrist and readmission within 30 days [13, 14, 36]. In those studies, similar results with our findings were shown. However, they were just analyzed in other countries, not South Korea. Thus, our results provide information that health policy makers and health care providers could use to reduce the readmission rate of psychiatric inpatients in South Korea.

Based on our findings, health policy makers should consider how to improve mental health care quality. Due to health care system characteristics like FFS, there are limited ways to control readmission [37]. Because health care providers receive payment for each treatment they provide, there is limited incentive to reduce readmission. As a result, inpatient readmission in South Korea has been difficult to control. However, our findings provide new evidence that health policy makers can use to revise policies. Our results suggest that the inpatient volume had an inverse association with patient`s outcome. We are not sure about the optimal values of inpatient volume per psychiatrist for maximizing the efficiency of psychiatric care, but there is a need to manage psychiatrist`s workload as appropriately. However, reducing inpatient volumes per psychiatrist mean either increasing psychiatry staffing or decreasing inpatient volume. Such strategies would accompany additional cost burden in psychiatric care. Therefore, health policy makers and decision makers have to first consider the tradeoff relationship between quality and cost, and then make efficient alternatives for improving psychiatric care in the near future. In addition, health policy makers should consider developing incentive programs to ensure a superior quality of care. Thus, such programs or alternatives would prevent inadequate psychiatric treatment and it could also be helpful in reducing the cost burden caused by adverse events such as readmission. This would eventually increase the accessibility of patients by reducing the cost barrier for using psychiatric care in South Korea, and then could improve the mental health of South Koreans.

Our study has several strengths compared to previous investigations. First, we used NHI claim data to analyze both patient- and hospital-level characteristics. Thus, our models reflect the diversity of patients and hospitals in South Korea, and the results will be helpful in establishing evidence-based health policies. Second, we considered hospital characteristics such as ownership status, teaching status, type of medical institution, and human resource availability. In previous studies on readmission, public hospitals have had higher readmission rates than private hospitals. However, we found higher readmission rates in private hospitals. We hypothesize that this is because private hospitals to occupy beds in order to make more profits, but such motivation was relatively less in public hospital based on ownership status [38]. Our results demonstrate that staff levels affect psychiatric inpatient care; in particular, the readmission rate is associated with the numbers of psychiatrists and pharmacists. This finding suggests that better quality care requires an appropriate balance of health care staff to deliver effective treatment. To test this hypothesis, it would be necessary to perform further analyses of detailed hospital data. Third, to our knowledge, this is the first study on the relationship between psychiatric inpatient readmission and inpatient volume per psychiatrist in South Korea, where readmissions are a major expense. Given the paucity of existing data, our results may prove helpful in managing South Korean mental health readmissions. Finally, our study employed LOS per psychiatrist to measure inpatient volume per psychiatrist. Thus, our results could be considered to provide more detailed inpatient volume per psychiatrist (as opposed to only examining patient volume).

Our study also has some limitations. First, substance use, an important indicator of mental health care quality, was not considered in our analysis because the relevant details were not included in our dataset. In addition, we only focused on five diagnostic groups, not all psychiatric disorders. As a result, these findings may not be generalizable to all psychiatric disorders. Also, we just consider repeat admission for same diagnosis rather than readmission due to any of the psychiatric diagnoses, because there were some limitations for accessibility of data. Next, we only analyzed cases with readmissions that took place in the same hospital. This was best way to include cross-hospital readmission in this study. However, we were unable to determine whether the each inpatient was hospitalized multiple times, as the data used in our study was based on only hospitalization cases, not inpatients details. In addition, the details about whether patients transferred to other hospitals could not be considered in this study, because such information were unavailable in the data used. Third, the NHI claim data we used included information from 53 hospitals. Thus, it may be difficult to generalize our results to South Korea as a whole, and we could not account for cases transferred from other hospitals. In addition, in the process of selecting the study population, we first performed the propensity score matching and excluded hospitals without hospitalization cases for the major five psychiatric disorders. Although it would be ideal to first exclude inappropriate hospitals which do not provide psychiatric care among total hospitals in South Korea and then perform the selection of the study population using the propensity score matching methods, there were limitations in conducting this as there are difficulties in accessing patient information due to issues such as ethics. Fourth, our results do not address cost because financial information was not included in our dataset. Also, our study could not assess the effects of comorbid mental disorders including depression. Fifth, the inpatient volume per psychiatrist as an outcome variable used in this study defined as an average of sum of LOS per each psychiatrist in each hospital. Therefore, we could not consider difference for workload each psychiatrist in each hospital by calculating method and limitation of data.

Despite these limitations, our results suggest that inpatient volume per psychiatrist substantially impacts the risk of psychiatric inpatient readmission within 30 days. These findings could be used by health policy makers and the government to identify potential solutions for controlling psychiatric inpatient readmission rates. However, further studies are needed to determine effective strategies to reduce psychiatric inpatient readmission rates.

## Conclusions

Psychiatric inpatient readmission rates within 30 days of release were lower in hospitals with lower inpatient volume per psychiatrist, especially for those patients hospitalized for “schizophrenia, schizotypal, and delusional disorders” and “mood disorders.” There need more further study which investigating more detailed relationship, so the health policy makers and hospital managers could reduce readmissions for psychiatric disorders and other diseases in the future.

### Ethics approval and consent to participate

Out study utilized secondary data reported on the aggregate level and each case in our dataset was converted to random number for avoiding identification. Thus, it was not necessary to obtain research ethics approval and informed consents from the patients.

### Consent for publication

Not applicable.

### Availability of data and materials

The data used in this study were obtained from the Health Insurance Claim data, it can only be disclosed to the people who had authorized.

## Abbreviations

ANOVAs: analysis of variances
CI: confidence interval
FFS: fee for service
GEE: generalized estimating equation
ICD: International classification of diseases groupings
LOS: length of stay
NHI: National Health Insurance
OECD: Organization for Economic Co-operation and Development
OR: odds ratio
SD: standard deviation
