# Erratum to: Readmission rates of South Korean psychiatric inpatients by inpatient volumes per psychiatrist

**DOI:** 10.1186/s12888-016-0899-1

**Published:** 2016-06-27

**Authors:** Kyu-Tae Han, Seo Yoon Lee, Sun Jung Kim, Myung-Il Hahm, Sung-In Jang, Seung Ju Kim, Woorim Kim, Eun-Cheol Park

**Affiliations:** Department of Public Health, Graduate School, Yonsei University, Seoul, Republic of Korea; Institute of Health Services Research, Yonsei University College of Medicine, Seoul, Republic of Korea; Department of Health Policy and Management, Graduate School of Public Health, Yonsei University, Seoul, Republic of Korea; Department of Health Administration and Management, Soonchunhyang University, Asan, Republic of Korea; Department of Preventive Medicine, Yonsei University College of Medicine, 50 Yonsei-ro, Seodaemun-gu, Seoul, 120-752 Republic of Korea

## Erratum

Following publication of the original article in *BMC Psychiatry* [1], it was brought to our attention that the results and conclusions sections in the abstract are incorrect:

- The results section in the abstract should read: Readmissions within 30 days accounted for 1,598 (4.5 %) claims. Multilevel analysis demonstrated that inpatient volume per psychiatrist were positively related with readmission within 30 days (low odds ratio [OR]: 0.38, 95 % confidence interval [CI]: 0.28–0.51; mid-low OR: 0.48, 95 % CI: 0.36–0.63; mid-high OR: 0.55, 95 % CI: 0.44–0.69; Q4 = ref). The subgroup analysis by diagnosis revealed that both “schizophrenia, schizotypal, and delusional disorders” and “mood disorders” had positive relationships with readmission risk for all volume groups.
- The conclusions section in the abstract should read: We observed a positive association between inpatient volume per psychiatrist and the 30-day readmission rate of psychiatric inpatients, suggesting that it could be a useful quality indicator in mental health care.

We apologize for the inconvenience this may have caused.
